# Mood instability as a transdiagnostic predictor of cannabis use in attention-deficit/hyperactivity disorder and depression: A natural language processing analysis of electronic health records from 13,025 adolescents

**DOI:** 10.1192/j.eurpsy.2025.10095

**Published:** 2025-08-22

**Authors:** Asilay Seker, Edward Bullock, Susie Chandler, Rashmi Patel, Diego Quattrone, Craig Colling, Edmund J. S. Sonuga-Barke, Johnny Downs

**Affiliations:** 1 CAMHS Digital Lab, King’s Maudsley Partnership, King’s College London and South London and the Maudsley NHS Foundation Trust, London, UK; 2 Department of Child and Adolescent Psychiatry, Institute of Psychiatry, Psychology & Neuroscience, King’s College London, London, UK; 3 Autism Research Centre, University of Cambridge, Cambridge, UK; 4 Department of Psychiatry, University of Cambridge, Cambridge UK; 5 National Institute for Health Research (NIHR) Biomedical Research Centre, South London and Maudsley NHS Foundation Trust, London, UK; 6 South London and the Maudsley NHS Foundation Trust, London, UK; 7 Social, Genetic and Developmental Psychiatry Centre, Institute of Psychiatry, Psychology and Neuroscience, King’s College London, London, UK; 8 Department of Biomedicine, Neuroscience and Advanced Diagnostics (BiND), Psychiatry Section, University of Palermo, Palermo, Italy

**Keywords:** ADHD, adolescent, cannabis, depression, emotion dysregulation, mood instability, natural language processing

## Abstract

**Background:**

Cannabis use is elevated in youth with depression and attention-deficit/hyperactivity disorder (ADHD), but drivers of this increase remain underexplored. The self-medication hypothesis suggests cannabis is used by patients for mood regulation, a common difficulty in ADHD and depression. This study aimed to examine associations between mood instability and cannabis use in a large, representative clinical cohort of adolescents diagnosed with ADHD and/or depression.

**Methods:**

Natural language processing (NLP) approaches were utilised to identify references to mood instability and cannabis use in the electronic health records of adolescents (aged 11–18 years) with primary diagnoses of ADHD (*n* = 7,985) or depression (*n* = 5,738). Logistic regression was used to examine mood instability as the main exposure for cannabis use in models stratified by ADHD and depression.

**Results:**

Mood instability was associated with a 25% higher probability of cannabis use in adolescents with ADHD compared to those with depression. Following adjustment for available sociodemographic and clinical covariates, mood instability was associated with increased cannabis use in both ADHD (aOR: 1.61 [95% CI: 1.41–1.84]) and depression (aOR: 1.38 [95% CI: 1.21–1.57]) groups.

**Conclusions:**

This was the first study to explore the differential impact of mood instability on adolescent cannabis use across distinct diagnostic profiles. NLP analysis proved an efficient tool for examining large populations of adolescents accessing psychiatric services and provided preliminary evidence of a link between mood instability and cannabis use in ADHD and depression. Longitudinal studies using direct measures or tailored NLP techniques can further establish the directionality of these associations.

## Introduction

Cannabis is the most commonly consumed recreational drug across the globe and in Britain, estimated to be used by 15.4% of youth in the UK [1]. Despite its widespread use, robust evidence implicates adolescent-onset cannabis exposure as a significant risk factor for multiple adversities including psychotic symptoms, delinquency, poorer educational and mental health outcomes, and subsequent illicit substance dependence [2–6]. Cannabis use is particularly common in those with psychiatric conditions such as depression and attention-deficit/hyperactivity disorder (ADHD), two of the most common diagnoses given in British Child and Adolescent Mental Health Services (CAMHS) [7–13]. To date, most studies investigating predictors of elevated cannabis use in those with psychiatric diagnoses have either focused on population-based samples or comparisons of clinical groups with nonclinical controls [9, 14–16]. While this approach is useful for establishing broad risk patterns, there is a lack of clinical research examining specific drivers of cannabis use across diagnostic profiles. Clinical studies comparing distinct diagnostic groups of prevalent conditions such as depression and ADHD are needed to identify transdiagnostic determinants of cannabis use in adolescents and support clinicians in more tailored risk assessment and monitoring in these populations.

One potential transdiagnostic predictor of cannabis use in depression and ADHD is mood instability. Much like cannabis use, mood instability, or the related concept of emotional dysregulation, is common in youth with depression or ADHD, predicting poor psychosocial outcomes [17–22]. Furthermore, existing evidence proposes potential links between mood instability and cannabis use: On the one hand, consistent with a *self-medication hypothesis*, cannabis users often report turning to this substance to cope with mood difficulties and negative emotional experiences, particularly if they have underlying mental health conditions [14, 23–25]. At the same time, previous studies confirm the detrimental impact of chronic cannabis use on mood [26, 27]. While this suggests a bidirectional relationship between mood difficulties and cannabis use, mood instability is characterised as an emotion dysregulation trait with temporal consistency and may therefore represent an underlying risk factor for cannabis use in adolescents [28–31].

Despite its links with low self-regulation, ADHD, and depression in adolescents, research exploring mood instability as a potential risk factor for substance use in these vulnerable groups remains limited and inconclusive [32–34]. As a result, we do not currently know if and to what extent mood instability influences cannabis use in adolescents with ADHD or depression. Given that ADHD and depression account for more than one-third of CAMHS diagnoses in the UK, understanding how common traits such as mood instability affect cannabis use within these populations has important individual-, family-, and service-level implications [35]. A key challenge in studying mood instability and cannabis use in clinical settings remains the barriers in collecting real-world clinical data, particularly in children and adolescents [36]. While there are many standardised tools to screen for such issues, these are not routinely employed in CAMHS. Yet rich, narrative information nested in clinical notes typically includes mood and substance-related difficulties. This means data about issues that are common but subject to poor standardised screening, such as mood instability or cannabis use, can be surfaced from electronic healthcare records (EHRs) via novel methods such as natural language processing (NLP), which structures free-text clinical notes into analysable data [37]. This concept has been confirmed in previous research in which NLP approaches were successfully used to extract mood instability and cannabis use from EHRs [38, 39].

To address gaps in clinical research, we aimed to explore mood instability as a transdiagnostic predictor of cannabis use in adolescents with a clinical diagnosis of depression and/or ADHD. To ensure clinical relevance and alignment with CAMHS care pathways [40], we compared the impact of mood instability on cannabis use between adolescents with depression and those with ADHD, rather than with nonclinical control groups. We examined the EHRs of adolescents aged 11–18 years and extracted moodinstability-related terms from clinical notes utilising an NLP approach to substitute for the lack of routinely collected measures. We tested the hypothesis that mood instability was associated with increased cannabis use in both diagnostic groups, adjusting for multiple individual and contextual confounders.

## Methods

### Study design and sample

A cross-sectional study was carried out using the South London and Maudsley NHS Foundation Trust (SLaM) Clinical Record Interactive Search (CRIS). CRIS was set up in 2008 to provide researchers with access to de-identified databases derived from its electronic health records, within a robust governance framework [41, 42]. CRIS currently holds anonymised health records of over 60,000 children and adolescents referred to SLaM CAMHS since 2007 [43]. The sample for this study consisted of all children and adolescents referred to SLaM CAMHS between January 1, 2008, and December 31, 2022.

Included participants comprised adolescents aged 11–18 years referred to SLaM CAMHS and had a diagnosis of depression (F32x or F33x) or ADHD (F90.xx) according to the International Statistical Classification of Diseases and Related Health Problems 10th edition (ICD-10) between 2008 and 2022 before their 18th birthday [44]. The index date was the recorded date of depression or ADHD (index diagnosis). To reflect clinical reality, we did not define a separate ADHD+depression group. Instead, each young person was assigned to the diagnostic group corresponding to their index date, emulating the case allocation to relevant CAMHS care pathways based on index diagnosis. The presence of co-occurring ADHD or depression was retained as a covariate for later adjustment (see *Statistical Analysis*). 13,025 participants were included in the final analysis.

### Measures

#### Primary outcome: cannabis use

Cannabis use was extracted from patient EHRs as a binary flag (absent/present), using a previously validated NLP method that identified free-text mentions referring to cannabis use. The method, based on a custom-built NLP software tool interfacing with CRIS called TextHunter, was described in detail elsewhere [45]. Using this approach identified cannabis use with 0.81 precision and 0.95 recall [45] and was used in previous EHR-based adolescent research [39].

#### Main exposure: mood instability

As there are no routinely collected standardized mood instability measures in SLaM CAMHS, these data were substituted from NLP outputs. Using another TextHunter-based NLP tool, mood instability terms were extracted from electronic health records within three months before/after the ADHD or depression diagnosis and coded as present/absent (*Supplement*). The accuracy of mood instability identified by this approach was previously validated in a cohort of mental health patients including young people aged 16–25 years, indicating a precision of 0.91 and recall of 0.73 [38].

### Clinical and sociodemographic variables

Other demographic variables and clinical data were extracted from structured and free-text fields within CRIS. These included gender, date of birth, ethnicity, socioeconomic status (based on neighborhood deprivation), number of contacts with CAMHS, and days of inpatient mental health admission (before/after the index date), and co-occurring psychiatric diagnoses. Medication use, including antidepressant, antipsychotic, hypnotic, and ADHD medications, was again extracted via previously developed NLP tools [46].

Age at index date was calculated by subtracting the date of birth from the index date. The follow-up duration was the period (in years) from the index date until either the last service use date, or 18th birthday, or the window end date, whichever came first. Ethnicity was recorded according to the defined UK Office for National Statistics (ONS) categories [47]. Nine ethnicity categories were collapsed into five to improve statistical sensitivity, which is consistent with prior research utilising CRIS [48, 49]. Neighbourhood deprivation was used as a proxy for socioeconomic status and measured using the multiple indices of deprivation (IMD) for small areas [50]. These indices incorporate various aspects of deprivation such as income, employment, education, health, crime, barriers to housing and services, and living environment, which are weighted differently and combined to form a comprehensive deprivation score.

The presence of multiaxial ICD-10 Axis II psychiatric diagnoses was extracted from structured data fields where available or substituted by outputs from previously validated NLP tools and recorded as binary variables [44, 51]. These included ADHD, depression, psychosis, eating disorders, obsessive-compulsive disorder (OCD), phobias, anxiety, intellectual disability, conduct disorder, and emotional disorders (*Supplement*). The pharmacological treatment provision including antipsychotic, antidepressant, hypnotic, and ADHD medication within 12 months of diagnosis was extracted from free text utilising NLP tools into a binary variable.

Service use was calculated by extracting the total number of appointments each service user attended at SLaM before and after the index date. Inpatient admission duration was determined based on the number of days spent in a SLaM CAMHS inpatient unit before and after the index date.

We also extracted scores from the Children’s Global Assessment Scale (CGAS), a widely used measure of overall psychosocial functioning for children and adolescents within a range of 1–100 points with lower scores indicating more impaired functioning, where available [52, 53]. As a patient can have multiple CGAS assessments, we coded the CGAS score closest to the index date as an indicator of baseline functioning and used the cutoff of 50 to group functioning levels.

### Statistical analysis

The data were analysed using Stata (V.18.0). Descriptive statistics for exposure, covariate, and outcome variables were reported as the mean for age at index date, CGAS scores, CAMHS use, and days in inpatient mental health admission, and as frequencies and percentages for all other categorical variables. To assess differences in sociodemographic and clinical characteristics based on cannabis use across groups, we used chi-square tests for categorical variables and Mann–Whitney U or t-tests for continuous variables, as appropriate (*Supplement*).

We tested the interaction between diagnostic group (ADHD, depression) and mood instability in predicting cannabis use and calculated predicted cannabis use probability controlling for this interaction in the logistic regression analysis. We also tested for interactions between gender and mood instability in relation to cannabis use. Following stratification by diagnostic groups of depression and ADHD, multivariable logistic regression was employed to examine the association between mood instability and cannabis use, adjusting for available sociodemographic and clinical covariates. We controlled for the effect of co-occurrence between ADHD and depression in the adjusted logistic regression model.

### Ethics and consent

CRIS was approved as an anonymised data resource for secondary analysis by the Oxfordshire Research Ethics Committee C (08/H0606/71 + 5). This study was approved under the National Institute of Health Research Maudsley Biomedical Research Centre CRIS oversight committee (Ref: 22-066).

## Results

### Descriptive characteristics

The final sample included 13,025 adolescents (mean age = 14.97 years [SD = 1.93]; 52.93% female). Cannabis use and mood instability were documented in 30 and 28.4% of the whole sample, respectively. Descriptive characteristics, stratified by index diagnosis, are presented in Table 1. The ADHD group was predominantly male, whereas the depression group had a higher proportion of females. In both groups, White was the most commonly recorded ethnic background, and autism was the most frequent co-occurring diagnosis. Compared to those with ADHD, adolescents in the depression group demonstrated more frequent use of CAMHS services with longer durations of inpatient admissions following diagnosis, and were more likely to fall within the lower baseline functioning category. Rates of both mood instability and cannabis use were also higher in the depression group.

**Table 1.** Sociodemographic and clinical characteristics of adolescents (*n* = 13,025)

	ADHD, *n* (%) 7,985(61.31)	Depression, *n* (%) 5,738(44.05)
Mood instability, *N*(%)	1,806(22.6%)	2,198(38.3%)
Cannabis use, *N*(%)	2,324(29.1%)	1,891(33.0%)
Gender, *N*(%)		
Male	4,695(59.2%)	1,668 (29.3%)
Female	3,235(40.8%)	4,019 (70.7%)
Age at diagnosis, mean(SD)	14.46(0.02)	15.72(0.02)
Follow-up time (years), mean(SD)	2.33(.03)	1.75(.03)
Ethnicity, *N*(%)^b^		
White	3,916 (49.1%)	2,923 (51.0%)
Black	1,528 (19.2%)	1,097 (19.1%)
Asian	329 (4.1%)	360 (6.3%)
Mixed	821 (10.3%)	539 (9.4%)
Not stated	1,024 (12.8%)	537 (9.4%)
Other	356 (4.5%)	278 (4.8%)
Neighbourhood characteristics, *N*(%)^c^		
1st (least deprived)	1,989(25.6%)	1,367(24.3%)
2nd	1,948(25.1%)	1,426(25.4%)
3rd	1,931(24.9%)	1,404(25.0%)
4th (most deprived)	1,894(24.4%)	1,418(25.3%)
Children’s Global Assessment Scale (CGAS) scores, *N*(%)		
0–50 (poor-to-moderate functioning)	2,715(34.0%)	2,555(44.5%)
51–100 (variable-to-superior functioning)	5,270(66.0%)	3,183(55.5%)
Service use, mean(SD)		
Prior to index date	6.39(0.19)	6.96(0.21)
Post index date	10.56(0.26)	17.73(0.45)
Inpatient admission, mean(SD)		
Prior to index date	0.56(0.08)	1.51(0.166)
Post index date	3.32(0.28)	10.56(0.26)
Co-occurring conditions, *N*(%)		
Autism spectrum disorder	3,304(41.4%)	985(17.2%)
ADHD	-	698(12.2%)
Depression	698(8.7%)	-
Psychosis	508(6.4%)	467(8.1%)
Eating disorders	256(3.2%)	382(6.7)
Obsessive compulsive disorder	265(3.3%)	207(3.6%)
Phobia	109(1.4%)	175(3%)
Anxiety	781(9.8%)	798(13.9%)
Conduct disorder	583 7.3%)	126(2.2%)
Emotional disorder	745(9.3%)	446(7.8%)
Tic disorders	112(1.4%)	15(0.3%)
Intellectual disability	596(7.5%)	95(1.7%)
Medications, *N*(%)		
ADHD medication	2,412(30.2%)	215(3.7%)
Antidepressant medication	777(9.7%)	1,637(28.5%)
Antipsychotic medication	642(8%)	343(6%)
Hypnotic medication	630(7.9%)	487(8.5%)

*Note:* ADHD: attention deficit hyperactivity disorder. Missing values: ^a^ = 95, ^b^ = 14, ^c^ = 327.

### Cannabis use outcome

Within the total sample of adolescents, mood instability was associated with a greater than two-fold increase in cannabis use (*OR*: 2.21[95%*CI* 2.04–2.40], *p* < 0.001). This association was sustained following adjustment for multiple potential confounders (*OR*:1.50[95%*CI* 1.36–1.65], *p* < 0.001) (Supplementary Table 2).

**Figure 1:**
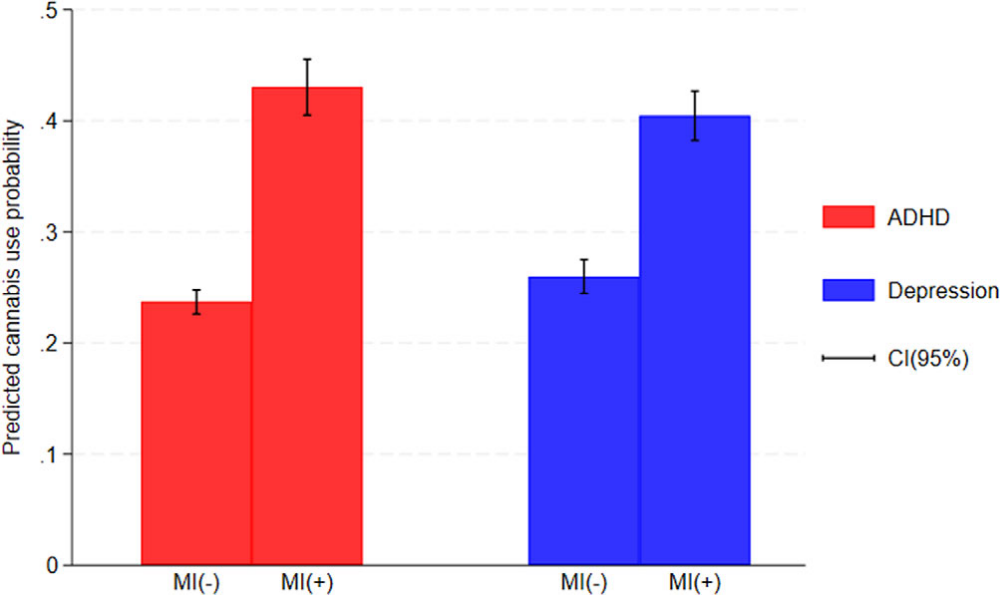
Predicted cannabis use probability, adjusted for interactions between NLP-identified mood instability and index diagnostic groups. ADHD: attention deficit hyperactivity disorder, CI: confidence interval, MI: mood instability.

For cannabis use, mood instability showed significant interaction between index diagnostic group (*OR*:.80[95%*CI* .68–.94], *p* = .009) and gender (*OR*:.77[95%*CI* .65–.91], *p* = .003) in the bivariate regression models. However, following adjustment for confounders, this effect modification was sustained only for diagnostic group (OR:.80[95%*CI* .66–.96], *p* = .017), while the interaction with gender was no longer significant (*OR*:.86[95%*CI* .71–1.03], *p = .118).* Probability predictions controlling for the interaction between mood instability and diagnostic group showed that mood instability was associated with an increased predicted probability of cannabis use in both ADHD (from .23[95% *CI*: .22–.24], *p* < 0.001 to .43[95% *CI*: .40–.45], *p* < 0.001) and depression groups (from .25[95% *CI*: .24–.27], *p* < 0.001 to .40[95% *CI*: .38–.42], *p* < 0.001) (Figure 1). This corresponded to a 25% higher likelihood of cannabis use due to mood instability in adolescents with ADHD, compared to those with depression ([95% *CI*: 1.06–1.49], *p* = 0.008).

Following stratification by diagnostic group, mood instability was again associated with increased cannabis use in both ADHD (*OR*: 2.49[95%*CI*: 2.23–2.75], *p* < 0.001) and depression (*OR*: 1.95[95%*CI*: 1.74–2.18], *p* < 0.001) groups, as shown in the univariate logistic regression analyses. After adjusting the model for available sociodemographic and clinical characteristics, mood instability was still associated with a 61% higher likelihood of cannabis use ([95%*CI*: 1.41–1.84], *p* < 0.001) in adolescents with ADHD and 38% in those with depression ([95%*CI*: 1.21–1.57], *p* < 0.001).

Table 2 presents the crude odds ratios and the fully adjusted logistic regression models for cannabis use for ADHD and depression diagnoses. Male adolescents were significantly more likely to use cannabis compared to their female peers. Among all recorded ethnicity categories, mixed ethnic background was the most strongly associated with increased cannabis use. Severe neighbourhood deprivation was also linked to increased cannabis use, but this was significant only in the ADHD group. Psychosis, conduct disorder, and emotional disorders were all associated with higher rates of cannabis use, regardless of the index diagnosis. ADHD medication use was another clinical factor significantly associated with increased cannabis use.

**Table 2.** Unadjusted and adjusted logistic regression models for cannabis use, stratified by ADHD and depression

	ADHD (*n* = 7,698)		Depression (*n* = 5,561)	
	OR (CI 95%)	aOR (CI 95%)	OR (CI 95%)	aOR (CI 95%)
Mood instability	2.49^**^(2.23–2.77)	1.61^**^(1.41–1.84)	1.95^**^(1.74–2.18)	1.38^**^(1.21–1.57)
Gender				
Female	Reference	Reference	Reference	Reference
Male	.95(.86–1.05)	1.25^**^(1.11–1.41)	1.26^**^(1.12–1.42)	1.41^**^(1.23–1.62)
Age at index date	1.20^**^(1.18–1.25)	1.28^**^(1.24–1.32)	1.07^**^(1.03–1.11)	1.18^**^(1.13–1.23)
Ethnicity				
White	Reference	Reference	Reference	Reference
Black	.83*(.73–.95)	.90(.77–1.04)	.76^**^(.65–.88)	.82*(.69–.97)
Asian	.56^**^(.43–.74)	.57^**^(.42–.79)	.43^**^(.33–.57)	.43^**^(.32–.58)
Mixed	1.21*(1.03–1.41)	1.33*(1.11–1.59)	1.25*(1.04–1.51)	1.42^**^(1.15–1.74)
Not stated	.52^**^(.44–.62)	.72^**^(.6–.87)	.45^**^(.36–.56)	.68*(.54–.87)
Other	.55^**^(.42–.71)	.60^**^(.44–.81)	.58^**^(.43–.76)	.59^**^(.43–.80)
Neighbourhood characteristics				
1st (least deprived)	Reference	Reference	Reference	Reference
2nd	1.07(.93–1.23)	1.14(.97–1.33)	1.08(.92–1.26)	1.17(.98–1.40)
3rd	1.02(.88–1.17)	1.19*(1.01–1.4)	.93(.80–1.1)	1.17(.97–1.40)
4th (most deprived)	1.07(.93–1.23)	1.17*(1.003–1.37)	.96(.82–1.12)	1.16(.97–1.39)
Children’s Global Assessment Scale (CGAS) scores				
0–50 (poor-to-moderate functioning)	Reference	Reference	Reference	Reference
51–100 (variable-to-superior functioning)	.49^**^(.44–.54)	.64^**^(.56–.72)	.65^**^(.58–.72)	.89(.78–1.01)
Service use				
Prior to index date	1.023^**^(1.019–1.026)	1.015^**^(1.011–1.019)	1.019^**^(1.014–1.023)	1.010^**^(1.005–1.014)
Post index date	1.02^**^(1.022–1.028)	1.02^**^(1.01–1.026)	1.015^**^(1.013–1.017)	1.014^**^(1.011–1.017)
Inpatient admission				
Prior to index diagnosis	1.04^**^(1.02–1.05)	1.01*(1.004–1.03)	1.02^**^(1.01–1.03)	1.006(.99–1.01)
Post index diagnosis	1.01^**^(1.012–1.019)	1.005^**^(1.002–1.008)	1.011^**^(1.009–1.013)	1.006^**^(1.004–1.008)
Co-occurring conditions				
Autism spectrum disorder	.73^**^(.66–.8)	.56^**^(.50–.64)	1.77^**^(1.54–2.03)	.85(.71–1.01)
Psychosis	3.40^**^(2.83–4.09)	2.08^**^(1.67–2.58)	2.80^**^(2.31–3.39)	1.79^**^(1.43–2.24)
Eating disorders	1.48*(1.14–1.9)	.62*(.45–.87)	1.56^**^(1.26–1.92)	.94(.73–1.21)
Obsessive compulsive disorder	.76(.57–1.02)	.33^**^(.23–.47)	1.01(.75–1.36)	.62*(.44–.87)
Phobia	.64(.40–1.02)	.42*(.24–.73)	.83(.59–1.15)	.52^**^(.35–.77)
Anxiety	1.25*(1.06–1.46)	.87(.72–1.05)	1.24*(1.06–1.45)	.82*(.68–.98)
Conduct disorder	2.17^**^(1.83–2.58)	1.95^**^(1.60–2.37)	2.78^**^(1.94–3.97)	2.07^**^(1.38–3.09)
Emotional disorder	1.80^**^(1.54–2.10)	1.36^**^(1.13–1.63)	1.68^**^(1.38–2.04)	1.27*(1.01–1.59)
Depression	2.06^**^(1.76–2.41)	1.05(.87–1.28)	-	-
ADHD	-	-	1.72^**^(1.46–2.02)	1.40^**^(1.14–1.72)
Tic disorders	1.01(.67–1.53)	.90(.57–1.44)	.50(.14–1.8)	.29(.07–1.12)
Intellectual disability	.63^**^(.51–.77)	.45^**^(.36–.58)	1.03(.67–1.58)	.56*(.34–.93)
Medications				
ADHD medication	1.82^**^(1.64–2.02)	1.57^**^(1.39–1.78)	2.42^**^(1.84–3.19)	1.41*(1.008–1.99)
Antidepressant medication	1.97^**^(1.69–2.29)	.92(.74–1.13)	1.91^**^(1.7–2.15)	1.10(.94–1.28)
Antipsychotic medication	1.88^**^(1.59–2.21)	.93(.74–1.17)	2.20^**^(1.77–2.74)	.96(.72–1.27)
Hypnotic medication	2.02^**^(1.71–2.38)	1.2(.96–1.49)	2.19^**^(1.82–2.64)	1.27*(1.01–1.60)

*Note:* *p < 0.05, ^**^p < =0.001, ADHD: attention deficit hyperactivity disorder, aOR: adjusted odds ratio.

## Discussion

To the best of our knowledge, this is the first study to specifically examine the links between mood instability and cannabis use in adolescents with clinically recognised depression and/or ADHD. As hypothesised, we found that mood instability was associated with increased cannabis use reported in adolescents with underlying ADHD and/or depression, even after accounting for a range of individual and contextual factors. While this is the first study of its kind to explore this association in the adolescent age group, previous research has also suggested that mood dysregulation predicts substance use, including cannabis [54–56]. Interestingly, our analyses revealed mood instability to have a stronger link with increased cannabis use in adolescents with ADHD compared to those with depression. While no previous studies have directly compared the influence of mood instability on cannabis use across psychiatric diagnoses, our findings align with earlier reports implicating emotion dysregulation as a key factor in the elevated use of this substance observed in individuals with ADHD [57, 58]. The higher rates of mood instability found in young people with depression, a mood disorder, compared to those with ADHD, a neurodevelopmental condition, are as expected and support previous reports from adult studies [59, 60]. Although we lack reference rates for mood instability in adolescents with ADHD, the frequency of mood instability found in our cohort is consistent with previous reviews on the prevalence of emotion dysregulation, of which mood instability is a component, in this group [61, 62]. Cannabis use rates in our cohort were higher than the population estimates, also consistent with the previous research reporting elevated cannabis use in those with ADHD and depression [8, 10]. Building on previous studies demonstrating the efficiency of NLP methods to identify poorly screened-for risk factors such as mood instability and cannabis use, our findings show NLP can be successfully applied to examine child mental health data [38, 63, 64]. While the mood instability NLP tool was originally developed for predominantly adult mental health records, prior work demonstrated its utility in young people aged 16–25 years, indicating validity in young patients [38]. Nonetheless, tailoring the algorithm to better capture CAMHS-specific documentation style and terminology is required to improve its sensitivity and precision in this population. As far as we are aware, this is one of the largest-scale clinical studies to date modeling cannabis use as an outcome in adolescents with psychiatric diagnoses, accounting for a broad range of sociodemographic and clinical factors. Therefore, our findings lay the groundwork for further research to explore mood instability as a potential target for interventions to reduce cannabis use and related adversity in at-risk adolescent groups with underlying depression or ADHD [56].

In addition to the associations between mood instability and cannabis use, our study profiled additional sociodemographic and clinical features of cannabis-using young people. Consistent with existing literature, we observed significantly higher odds of cannabis use among males in our sample [59, 65]. However, the absence of a significant interaction between gender and mood instability in the adjusted model suggests that the effect of mood instability on cannabis use is not moderated by gender once other relevant patient characteristics are accounted for. Although the research examining the ethnic determinants of cannabis use among adolescents is limited, our findings align with the UK Drug Policy Commission review, where individuals from mixed ethnic backgrounds had significantly higher levels of cannabis use compared to other ethnic groups, and younger age was noted to drive this increase [47, 66]. Mixed ethnic groups were again reported to have the highest cannabis use prevalence among ethnicity categories, based on the Crime Survey England & Wales [66]. Albeit less recent, the Adult Psychiatric Morbidity Survey found the highest cannabis use rates in the Black population, followed by mixed ethnic groups [66]. While these differences could relate to the younger mean age of our clinical sample and the changing trends in cannabis use [67], they could also stem from our data source: Our analyses relied on electronic health records, and as shown in a recent review, diagnosis and recording of clinical problems may be influenced by racial biases [68]. Future research may benefit from qualitative methods for enhanced depth of exploration to understand the potential role of racial biases in eliciting and documenting substance use in young people.

Another finding of note was that the odds of cannabis use was increased in young people who received their index ADHD or depression diagnosis at a later age, and this was more pronounced in the ADHD group. The mean age at ADHD diagnosis, a neurodevelopmental condition that typically manifests from early childhood, for our cohort was considerably older than global averages [69]. While this may partly reflect the focus of our study on adolescence, it also suggests delays in timely diagnosis. Increased substance use has previously been reported as a potential risk related to long waiting times for mental health support [70, 71]. In accordance with previous research, our findings suggest these delays in access to timely assessments and care may exacerbate maladaptive coping behaviours such as cannabis use, particularly in vulnerable groups including adolescents with ADHD.

We found that those in our sample receiving ADHD medication were at higher odds of cannabis use. Existing literature suggests ADHD medication, particularly if started at an early age, may be protective against later substance use [72–74]. While our study design was not able to confirm any direction of causality between ADHD medications and cannabis use, the interpretation of our findings should be grounded in clinical context in which strong confounding by indication is likely, rather than any iatrogenic exacerbation of substance use with ADHD medication: British guidelines recommend ADHD medication only for those with persisting significant impairment, and pharmacotherapy is often reserved for young people with moderate-to-severe ADHD [75]. Indeed, receiving ADHD medication should therefore be considered a proxy for the severity of ADHD-related impairment in our sample. As such, these findings corroborate previous evidence linking ADHD symptom severity with increased substance use [57, 76–78].

### Strengths and limitations

A notable strength of this study was its substantial sample size of 13,025, derived from the electronic health records of one of Europe’s largest mental health service providers, covering a highly diverse and representative population [41]. The database used in this study, CRIS, is not only one of the most comprehensive of its kind, but it also offers a rare depth of clinical information that goes far beyond traditional case registries [79]. CRIS provides a highly detailed profile of patients through clinical notes, enabling extraction of fine-grained patient insights and data linkages to fill in gaps in patient records [41]. Our design, therefore, maximised the representativeness of our participants, who were not filtered by research criteria, and reflected the everyday clinical practice, thereby enhancing generalisability and impact. We also demonstrated the clinical utility of automated NLP approaches to identify mood instability in adolescent EHRs, which enabled extraction and analysis of extensive data while minimising potential human error. The literature definition of mood instability lacks precision; however, it is described as remaining fairly stable over time [28, 31]. Although bidirectional associations are possible, this temporal consistency supports the clinical plausibility of our model, which is the first attempt to investigate mood instability as a predictor of cannabis use. This was also the first study, to our knowledge, to compare the impact of mood instability on cannabis use in adolescents with ADHD and/or depression. Future studies should aim to replicate these findings to ascertain the distinct effect of mood instability on cannabis use in adolescents with ADHD and to identify the underlying mechanisms driving this association, which could inform the development of more precise interventions.

This study also had limitations, which should be considered when interpreting the findings and planning future research. Data extraction using NLP algorithms applied to electronic health records relies on clinician-documented findings. While this design allows the inclusion of participants who may otherwise experience barriers to participation in clinical research [38], it also means the presence of mood instability or cannabis use could not be detected unless recorded by clinicians. We extracted both mood instability and cannabis use as binary flags (present/absent); therefore, we could not examine the impact of severity or frequency for either of the issues. Future research could address these limitations by utilising NLP approaches developed specifically for CAMHS records and incorporating additional features to further contextualise the symptom of interest.

## Conclusions

We identified significant associations between mood instability and cannabis use in adolescents with ADHD and/or depression, two of the most commonly diagnosed conditions in CAMHS. The concerning link between older age at diagnosis and cannabis use underscores the importance of timely psychiatric assessment and care for adolescents. Our study also provided an example of clinical record analysis using NLP methods to efficiently examine mood instability and cannabis use in a large, representative cohort of adolescents, yielding clinically relevant findings. Further refinement of NLP data-surfacing methods can maximise the use of routinely collected clinical data to understand the impact of transdiagnostic risk factors, such as mood instability, on adverse outcomes including substance use. Longitudinal studies utilising direct measurements in clinically recruited adolescents could help confirm the directionality of these associations.

## Supporting information

10.1192/j.eurpsy.2025.10095.sm001 Seker et al. supplementary material Seker et al. supplementary material

## Data Availability

The data that support the findings of this study are available from SLaM, but restrictions apply to the availability of these data, and they are not publicly available.

## Supplementary material

The supplementary material for this article can be found at http://doi.org/10.1192/j.eurpsy.2025.10095.
