# Supplementary material for: Mood instability as a transdiagnostic predictor of cannabis use in attention-deficit/hyperactivity disorder and depression: A natural language processing analysis of electronic health records from 13,025 adolescents
Source: Eur Psychiatry. 2025 Aug 22;68(1):e139. doi: 10.1192/j.eurpsy.2025.10095 (PMC12538179; doi:10.1192/j.eurpsy.2025.10095)
Supplement: Seker et al. supplementary material [file S0924933825100953sup001.docx]

Supplementary Table 1. Sociodemographic and clinical characteristics of adolescents with ADHD or depression based on their cannabis use (n=13,025)

|  | ADHD, n(%)  7,985(61.31) | | Depression, n(%)  5,738(44.05) | |
| --- | --- | --- | --- | --- |
|  | **Cannabis use (-), n(%)**  **5,661(70.9)** | **Cannabis use (+)**, **n(%)**  **2,324(29.1)** | **Cannabis use (-)**, **n(%)**  **3,847(67.04)** | **Cannabis use (+)**,  **n(%)**  **1,891(32.96)** |
|  |  |  |  |  |
| Mood instability, N(%) | 998(17.63)** | 808(34.77) | 1,270(33.01)** | 928(49.07) |
| Gender, N(%)(**)^a^ |  | | | |
| Male | 3,351(59.51) | 1,344(58.46) | 1,057(27.70)** | 611(32.66) |
| Female | 2,280(40.49) | 955(41.54) | 2,759(72.30)** | 1,260(67.34) |
| Age at diagnosis, mean(SD) | 14.24(.02)** | 14.99(.03) | 15.66(.02)** | 15.84(.03) |
| Follow-up time (years), mean(SD) | 2.47(.02) | 2.44(.03) | 2.05(.02) | 1.98(.03) |
| Ethnicity, N(%)^b^ |  |  |  |  |
| White | 2,668(47.18)** | 1,248(53.82) | 1,851(48.15)** | 1,072(56.72) |
| Black | 1,098(19.42) | 430(18.54) | 761(19.8) | 336(17.78) |
| Asian | 260(4.60)** | 69(2.98) | 287(7.47)** | 73(3.86) |
| Mixed | 524(9.27)** | 297(12.81) | 312(8.12)** | 227(12.01) |
| Not Stated | 822(14.54)** | 202(8.71) | 425(11.06)** | 112(5.93) |
| Other | 283(5.00)** | 73(3.15) | 208(5.41)* | 70(3.70) |
| Neighborhood Characteristics, N(%)^c^ |  | | | |
| 1^st^ (Least Deprived) | 1,422(25.93) | 567(24.90) | 914(24.31) | 453(24.42) |
| 2^nd^ | 1,364(24.87) | 584(25.65) | 928(24.68) | 498(26.85) |
| 3^rd^ | 1,372(25.01) | 559(24.55) | 958(25.48) | 446(24.04) |
| 4^th^ (Most Deprived) | 1,327(24.19) | 567(24.90) | 960(25.53) | 458(24.69) |
| Children’s Global Assessment Scale (CGAS) scores, N(%) |  | | | |
| 0-50 (poor to moderate functioning) | 1,657(29.27)** | 1,058(45.52) | 1,579(41.04)** | 976(51.61) |
| 51-100 (variable to superior functioning) | 4,004(70.73)** | 1,266(54.48) | 2,268(58.96)** | 915(48.39) |
| Service Use, mean(SD) |  | | | |
| Prior to index date | 4.57(.15)** | 10.80(.55) | 5.50(.17)** | 9.92(.53) |
| Post index date | 7.26(.18)** | 18.60(.78) | 12.56(.34)** | 28.24(1.15) |
| Inpatient admission, mean(SD) |  | | | |
| Prior to index date | .12(.03)** | 1.63(.29) | .69(.11)** | 3.18(.44) |
| Post index date | 1.11(.17)** | 8.72(.87) | 21.79(1.50)** | 4.57(.40) |
| Co-occurring conditions, N(%) |  | | | |
| Autism Spectrum Disorder | 2,465(43.54)** | 839(36.10) | 552(14.35)** | 433(22.90) |
| ADHD | - | - | 390(10.14)** | 308(16.29) |
| Depression | 390(6.89)** | 308(13.25) | - | - |
| Psychosis | 223(3.94)** | 285(12.26) | 207(5.38)** | 260(13.75) |
| Eating Disorders | 160(2.83)* | 96(4.13) | 219(5.69)** | 163(8.62) |
| Obsessive Compulsive Disorder | 201(3.55) | 64(2.75) | 138(3.59) | 69(3.65) |
| Phobia | 86(1.52) | 23(0.99) | 124(3.22) | 51(2.70) |
| Anxiety | 520(9.19)* | 261(11.23) | 501(13.02)* | 297(15.71) |
| Conduct Disorder | 317(5.60)** | 266(11.45) | 54(1.40)** | 72(3.81) |
| Emotional Disorder | 439(7.75)** | 306(13.17) | 249(6.47)** | 197(10.42) |
| Tic Disorders | 79(1.40) | 33(1.42) | 12(0.31) | 3(0.16) |
| Intellectual Disability | 470(8.30)** | 126(5.42) | 63(1.64) | 32(1.69) |
| Medications, N(%) |  | | | |
| ADHD medication | 1,493(26.37)** | 919(39.54) | 100(2.60)** | 115(6.08) |
| Antidepressant medication | 443(7.83)** | 334(14.37) | 924(24.02)** | 713(37.70) |
| Antipsychotic medication | 371(6.55)** | 271(11.66) | 169(4.39)** | 174(9.20) |
| Hypnotic medication | 354(6.25)** | 276(11.88) | 243(6.32)** | 244(12.90) |

**p<0.05, **p<=0.001, ADHD: Attention Deficit Hyperactivity Disorder*. *Missing values: ^a^=95, ^b^=14, ^c^=327*

Supplementary Table 2. Unadjusted and adjusted logistic regression models for cannabis use (whole sample)

|  | **OR(CI 95%)** | **aOR(CI 95%)**  **(n=**12,591) |
| --- | --- | --- |
| **Mood Instability** | 2.21**(2.04-2.40) | 1.50**(1.36-1.65) |
| **Primary diagnosis** |  |  |
| ADHD | Reference | Reference |
| Depression | 1.28**(1.19-1.38) | .83**(.75-.93) |
| **Gender** |  |  |
| Male | Reference | Reference |
| Female | .98(.90-1.05) | .75**(.68-.82) |
| **Age at index date** | 1.16**(1.14-1.19) | 1.24**(1.21-1.27) |
| **Ethnicity** |  |  |
| White |  | Reference |
| Black | .80**(.72-.88) | .85*(.76-.96) |
| Asian | .53**(.43-.64) | .51**(.41-.64) |
| Mixed | 1.21*(1.07-1.37) | 1.38**(1.21-1.59) |
| Not Stated | .50**(.43-.57) | .71**(.61-.83) |
| Other | .55**(.45-.68) | .61**(.49-.76) |
| **Neighbourhood Characteristics** |  |  |
| 1^st^ (Least Deprived) | Reference | Reference |
| 2^nd^ | 1.06(.95-1.18) | 1.12*(1.0002-1.27) |
| 3^rd^ | .97(.87-1.09) | 1.16*(1.02-1.31) |
| 4^th^ (Most Deprived) | 1.02(.92-1.14) | 1.14*(1.01-1.29) |
| **Children’s Global Assessment Scale (CGAS) scores** |  |  |
| 0-50 (poor to moderate functioning) | Reference | Reference |
| 51-100 (variable to superior functioning) | .55**(.51-.60) | .74**(.68-.81) |
| **Service Use** |  |  |
| Prior to index date | 1.021**(1.018-1.024) | 1.013**(1.01-1.017) |
| Post index date | 1.019**(1.017-1.021) | 1.018**(1.015-1.02) |
| **Inpatient admission** |  |  |
| Prior to index diagnosis | 1.029**(1.022-1.035) | 1.008*1.002-1.01) |
| Post index diagnosis | 1.012**(1.011-1.014) | 1.006**(1.004-1.008) |
| **Co-occurring conditions** |  |  |
| Autism Spectrum Disorder | .91*(.83-.98) | .66**(.59-.73) |
| Psychosis | 3.10**(2.70-3.56) | 1.93**(1.64-2.27) |
| Eating Disorders | 1.49**(1.25-1.76) | .76*(.61-.93) |
| Obsessive Compulsive Disorder | .87(.70-1.07) | .44**(.34-.57) |
| Phobia | .80(.60-1.05) | .47**(.34-.66) |
| Anxiety | 1.22**(1.09-1.37) | .84*(.74-.97) |
| Conduct Disorder | 2.24**(1.91-2.62) | 2.11**(1.76-2.52) |
| Emotional Disorder | 1.69**(1.49-1.93) | 1.38**(1.19-1.60) |
| Tic Disorders | .96(.64-1.42) | .85(.55-1.33) |
| Intellectual Disability | .62**(.51-.75) | .47**(.38-.59) |
| **Medications** |  |  |
| ADHD medication | 1.57**(1.43-1.72) | 1.59**(1.41-1.78) |
| Antidepressant medication | 1.91**(1.74-2.11) | 1.009(.89-1.14) |
| Antipsychotic medication | 1.98**(1.72-2.27) | .98(.81-1.17) |
| Hypnotic medication | 2.07**(1.82-2.36) | 1.23*(1.04-1.45) |

**p<0.05, **p<=0.001, ADHD: Attention Deficit Hyperactivity Disorder, aOR: Adjusted Odds Ratio*

Search terms for Mood Instability Natural Language Processing application:

Chang* [0-2 words in between] *mood*

Extremes [0-2 words in between] *mood*

Fluctuat* [0-2 words in between] *mood*

Instability [0-2 words in between] *mood*

*labil* [0-2 words in between] mood

Rapid cycling [0-2 words in between] mood

*swings* [0-2 words in between] mood

*unpredictable* [0-2 words in between] mood

unsettled [0-2 words in between] mood

unstable [0-2 words in between] mood

*variable* [0-2 words in between] mood

*variation* [0-2 words in between] mood

*volatile* [0-2 words in between] mood

mood [0-2 words in between] chang*

mood [0-2 words in between] Extremes

mood [0-2 words in between] fluctuat*

mood [0-2 words in between] Instability

mood [0-2 words in between] *labil*

mood [0-2 words in between] Rapid cycling

mood [0-2 words in between] *swings*

mood [0-2 words in between] *unpredictable*

mood [0-2 words in between] Unsettled

mood [0-2 words in between] Unstable

mood [0-2 words in between] *variable*

Psychiatric diagnoses and corresponding ICD-10 codes:

Autism Spectrum Disorder (ICD-10 F84.0-F84.9)

Pyschosis (ICD-10 F1x.5, F20–F29, F31, F32.3, F33.3)

Eating Disorder (ICD-10 F50)

Obsessive-Compulsive Disorder (ICD-10 F42)

Phobia (ICD-10 F40.0-F40.9)

Anxiety (ICD-10 F41.0-F41.9)

Intellectual Disability (ICD-10 F70.0-F70.9, F71.0-F71.9, F72.0-F72.9, F73.0-F73.9, F78.0-F78.9, F79.0-F79.9)

Conduct Disorder (ICD-10 F91.0-F91.9, F92.0-F92.9)

Emotional Disorder (ICD-10 F93.0-F93.9)

Tic Disorders (ICD-10 F95.0-F95.9)
